# Fatness but Not Fitness Relative to the Fat-Free Mass Is Related to C-Reactive Protein in 18 Year-Old Adolescents

**DOI:** 10.1371/journal.pone.0130597

**Published:** 2015-06-15

**Authors:** Gunnhildur Hinriksdóttir, Ágústa Tryggvadóttir, Anna Sigríður Ólafsdóttir, Sigurbjörn Árni Arngrímsson

**Affiliations:** Center for Sport and Health Sciences, University of Iceland, Laugarvatn, Iceland; University of the Balearic Islands, SPAIN

## Abstract

**Introduction:**

The interaction between fatness, fitness, and C-reactive protein (CRP) in adolescents is not well characterized but may be important to prevent low grade inflammation. The purpose of this study was to assess the relationship between adiposity, different expressions of fitness, and CRP in late adolescence using direct measures of fitness and fatness.

**Methods:**

Anthropometric measurements were taken on 245 eighteen-year-old participants (116 girls). Fasting CRP, glucose, and insulin were measured and homeostatic model assessment (HOMA) calculated. Body composition was estimated via dual energy X-ray absorptiometry. Fitness was assessed with maximal oxygen uptake (VO_2_max) during a treadmill test and also expressed relative to the fat-free mass (VO_2_max_FFM_).

**Results:**

Prevalence of overweight/obesity based on body mass index (BMI) was 20.7% and 25.6% among girls and boys, respectively (p = 0.407), but 42.5% and 58.1% when based on body fat percentage (%fat, p = 0.015). Higher proportion of boys (81.3%) than girls (54.5%) were highly fit (p<0.001), but the percentage of girls with high levels of CRP was greater (12.1% vs 6.2%, p = 0.028). Adiposity, indicated with BMI, waist circumference, fat mass, android fat mass (aFM), or %fat, was positively associated with CRP independent of VO_2_max (r = 0.13-0.18, p<0.05) and VO_2_max_FFM_ (r = 0.24-0.32, p<0.001). VO_2_max, was negatively associated with CRP independent only of BMI and waist circumference (r = -0.21, p = 0.001), but not %fat, fat mass or aFM (r = -0.08 to -0.12, p>0.05). VO_2_max_FFM_ was unrelated to CRP with (r = -0.07 to -0.11, p>0.05) or without (r = -0.10, p = 0.142) adjustment for adiposity. Additional adjustment for HOMA did not change any of the relationships, although the coefficients were attenuated.

**Conclusions:**

Fatness has a greater association with CRP than fitness in late adolescence. However, VO_2_max_FFM_, which is truly independent of adiposity, is unrelated to CRP, indicating that the effects of fitness might be mediated via the fatness component embedded in fitness expressed relative to body mass.

## Introduction

Obesity has increased tremendously over the past decades and is one of the biggest health burdens in the world [1]. Iceland is no exception and studies have shown an increased rate of overweight and obesity in both children and adults [2,3]. Aerobic fitness (fitness) is another important health marker and is associated with lower rates of morbidity and mortality in adults [4,5] as well as various health outcomes in children and adolescents [6]. Moreover, adolescent fitness is negatively related to adult body fatness [7]. Unfortunately, some studies suggest that fitness among children and adolescents has declined over the last decades [8–10].

C-reactive protein (CRP) is a well-known marker of whole body systemic low-grade inflammation and data suggest that CRP is implicated in the development of cardiovascular disease [11]. Higher levels of CRP are positively associated with measures of adiposity [12–21] whereas better fitness is associated with lower CRP in children as well as adults [12–14,18–20,22,23]. The interactive effects of body fatness and fitness on CRP, however, have not been studied extensively. Some studies suggest that adiposity has a greater impact on CRP than fitness [12,14,19,23], whereas other suggest an independent association of fitness with CRP [13,17,22]. To further complicate this issue, the interactive effects may depend upon the age of the population studied as the independent relation of fitness to CRP appears less evident in children [12,14,16,17,23], which might be because CRP reflects chronic systematic low-grade inflammation that emerges over time [16]. Some intervention studies indicate that a decrease in CRP after an exercise intervention is dependent upon body mass or fat loss [21,24], others report that the effects of exercise are independent of changes in fatness and fitness [25], or even that changes in CRP are related to changes in physical activity and not body composition [26]. Not many intervention studies have investigated the fitness fatness interaction specifically and almost all of them have been conducted in middle-aged or older adults [20,27,28]. The results are conflicting, ranging from no relation between changes in CRP and changes in adiposity or fitness [20] to independent associations of changes in both fitness and fatness with changes in CRP [27]. In addition, many studies have used indirect measures or estimates of adiposity [12–14,16,22,26,27] and fitness [12–14,16,19,25–29], which may affect the nature of the relationship between fitness, fatness and CRP. Understanding the interrelationship of fitness and fatness may be the main way to develop preventive actions to attenuate systemic low grade inflammation [14] but precise measurements of the variables are needed [13].

The relation between fitness and CRP may be confounded or mediated by fatness, or fitness and fatness might share the same causal pathways [17]. In addition, fitness is traditionally expressed relative to body mass and is highly correlated with fatness leading to statistical problems due to multicolinearity, which can lead to wrong conclusions. Furthermore, since adiposity is a factor in both the value of fitness expressed relative to body mass and in value of the fatness measure controlled for (as is commonly done), the variables violate the statistical assumption of independence. Using a different approach by expressing fitness relative to the fat-free mass (FFM, fitness_FFM_) enables researchers to estimate the effect of fitness adjusted for body composition on CRP. This expression of fitness (fitness_FFM_) may be the best indirect size-independent estimate of the metabolic capacity of the muscle [30], and adiposity is not a component of fitness_FFM_. In contrast, the traditional way of expressing fitness relative to body mass is heavily influenced by adiposity, and absolute values for fitness such as L/min are greatly impacted by body size.

The primary purpose of this study was to assess the relationship between adiposity measurements, fitness and CRP in late adolescence, using direct measures of fitness and fatness. We used two different methods to distinguish the association of fitness from the association of adiposity; a) control for adiposity and expressing fitness relative to body mass (for comparative purposes as this is the usual practice) and b) expressing fitness relative to FFM. To our knowledge, the relation of fitness_FFM_ to CRP has not been studied previously. A secondary objective was to determine whether fatness or fitness had a stronger association to CRP in this relatively understudied population. We hypothesized that 1) both fatness and fitness would be independently associated to CRP although the relation between fatness and CRP would be stronger, 2) fitness_FFM_ would be more strongly related to CRP than fitness, and 3) overweight adolescents with high fitness levels would have lower levels of CRP than overweight adolescents with low fitness levels.

## Methods

### Study design and participants

Participants in this cross-sectional study were 18 year-old (17.7–18.9 yr) high-school students. To ensure a diverse group of participants, three high-schools in metropolitan Reykjavik were selected that had different academic structures offering both traditional academic studies and vocational education. Approximately 67% of all high-school students in Iceland attend high schools in metropolitan Reykjavik. In an attempt to get a final sample of approximately 300 participants, a total of 383 students were randomly selected (computerized random number generator) from the class registers and invited to participate in the study. Of those, 275 participated (73% and 69% participation among students in traditional academic studies and vocational education, respectively) and 250 participants completed the blood draw and were included in the analysis for this study. Those 25 who elected not to undergo the blood draw, did not differ from those who did on any body composition or fitness variable. Prior to participating in the study, which was approved by the National Bioethics Committee in Iceland VSNb2007110010/03-1, all participants signed an informed consent form along with a parent or legal guardian for those students who had not yet reached 18 years of age.

### Study protocol

Anthropometric measurements were taken in the morning at the participating schools. On another day (both mornings and afternoons), aerobic fitness was measured in the laboratory. The dual energy x-ray absorptiometry (DXA) (GE Lunar IDXA software 11.40.004; Little Chalfont, UK) measurements took place on the third day in the morning at the Icelandic Heart Association. Finally, on the fourth day in the morning, fasting blood samples were collected at the participating schools. Each participant finished all measurements in a span of 7–10 days.

### Body composition

Height was measured to the nearest 0.1 cm (Seca 206; Hamburg, Germany) and body mass to the nearest 0.1 kg (Seca 799) with the participants lightly dressed (i.e. underwear), and body mass index (BMI) calculated. Waist circumference (WC) was measured to the nearest 0.1 cm at the narrowest place between the lowest rib and the iliac crest at the end of normal exhalation with a spring loaded inelastic measuring tape. Body composition was assessed via DXA, which returned values for whole body fat mass, FFM, trunk fat mass, android fat mass (aFM) and body fat percentage (%fat).

### Aerobic fitness

Maximal oxygen uptake (VO_2_max) was assessed using open circuit spirometry (Parvomedics Trumax 2400; Sandy, UT, USA) with a graded exercise test protocol on a treadmill (Quasar med, HP Cosmos; Traunstein, Germany). Participants ran until exhaustion at a constant pace (8–13 km/hour depending upon fitness level) on the treadmill with a 2% grade increase every two minutes. Expired gases were sampled every 30 seconds, and heart rate (Polar heart rate monitor; Kempele, Finland) and rating of perceived exertion (Borg scale 6–20) were recorded every two minutes. A subject was considered to have reached VO_2_max if the oxygen uptake increased less than half of the estimated increase (based on the speed and grade [31]) between the last and next-to-last stage. If a participant did not reach VO_2_max based on that criterion, the test was still considered maximal if the subject reached at least two of the following three criteria: Respiratory exchange ratio ≥ 1.1; rating of perceived exertion ≥ 19; heart rate within 10 beats of age-dependent maximal heart rate (208–0.7*age) [32–35]. The maximal oxygen uptake relative to the FFM (VO_2_max_FFM_) was also calculated.

### Blood variables

Blood samples were drawn by venipuncture from the antecubital fossa of the non-dominant arm after an overnight fast (12 hours minimum) and after refraining from physical activity (other than ambulatory physical activity) for 12 hours. The blood was centrifuged and the serum drawn off and stored at -80°C until analysis. Serum high-sensitivity CRP, insulin, and glucose were assessed by the Landspitali University Hospital and homeostatic model assessment (HOMA) was determined by multiplying insulin with glucose and dividing by 22.5 [36].

### Statistical analysis

Statistical analysis was performed using SPSS statistical software, version 17.0 for Windows. The data was inspected for normality and outliers. Five participants (four girls, one boy) with CRP values above 10.0 mg^.^L^-1^ were determined outliers and excluded from further analysis [37]. CRP, HOMA, body mass, BMI, WC, fat mass, trunk fat mass, aFM and %fat were positively skewed and were normalized by logarithmic transformations. Independent samples t-test was used to assess sex differences and chi-square to assess proportional sex difference in body compositional, fitness, or CRP classifications. Partial correlations controlled for sex and subsequently HOMA (because of the known relation of HOMA to CRP, fitness and adiposity [14]) were used to assess the relationship between a) CRP and adiposity measurements (WC, BMI, fat mass aFM, and %fat) while controlling for VO_2_max, VO_2_max_FFM_; and b) CRP and VO_2_max or VO_2_max_FFM_ while controlling for the adiposity measures (one at a time).

Participants were classified based on VO_2_max into low (<42.2 and <35.5 ml/kg/min), average (42.2–45.69 and 35.5–39.49 ml/kg/min), or high (≥45.7 and ≥39.5 ml/kg/min) fitness categories for males and females, respectively [31]. The participants were also classified based on VO_2_max_FFM_ but because no empirical classifications for fitness_FFM_ exist, they were proportionally (within sex) classified in accordance with the VO_2_max classifications; i.e. the same percentage (within sex) as was within each VO_2_max category was classified into low (<56.3 and <55.1 ml/kg_FFM_/min), average (56.3–59.39 and 55.1–57.59 ml/kg_FFM_/min), or high (≥59.4 and ≥57.6 ml/kg/min) fitness_FFM_ categories for males and females, respectively. Additionally, participants were classified based on BMI into normal weight (<25 kg/m^2^) or overweight/obese (≥25 kg/m^2^) categories [38]. Likewise, participants were categorized according to Lohman et al. [39] on %fat into normal fat (<17.5% and <31.5%) and over-fat (≥17.5% and ≥31.5% body fat) categories for males and females, respectively. Based on these classifications, four fit-fat groups were created: low-fit/normal-weight, low-fit/overweight, high-fit/normal-weight, and high-fit/overweight (subjects classified in the average fitness/fitness_FFM_ category (n = 36) were omitted). Two-way ANOVA controlled for sex was used to examine the interaction between the fitness and fatness categorical classification. One-way ANOVA controlled for sex was used to detect differences between the four fit-fat groups with Post-Hoc comparisons using the modified Bonferroni adjustment to account for the family wise error rate. Group differences and relationships were determined significant at α-level < 0.05.

## Results

A total of 245 students (116 girls, 129 boys) were included in the final analysis. Significant sex differences (p < 0.01) were observed for all variables except BMI, CRP, and HOMA. Girls had more fat based on all DXA measures of fatness and lower fitness levels, whereas boys were taller, heavier, had greater WC, and more FFM (Table 1).

**Table 1:** Subject characteristics.

	Girls	Boys	Total
*Anthropometry*	*n* = 116	*n* = 129	*n* = 245
Height (cm)	168.1±5.6	182.2±6.5^†^	175.5±9.3
Body mass (kg)	63.7±9.9	77.7±14.3^†^	71.1±14.2
BMI (kg/m^2^)	22.6±3.5	23.4±4.2	23.0±3.9
Waist circumference (cm)	75.8±8.3	82.5±10.9^†^	79.3±10.3
*Body composition*	*n* = 113	*n* = 129	*n* = 242
Fat-free mass (kg)	43.3±4.7	61.4±7.8^†^	53.0±11.1
Fat mass (kg)	19.9±6.5	16.7±9.7^†^	18.2±8.5
Trunk fat mass (kg)	8.7±3.5	7.9±5.6^†^	8.3±4.8
Android fat mass (kg)	1.4±0.7	1.3±1.2*	1.4±1.0
Body fat (%)	31.0±5.8	20.4±7.7^†^	25.3±8.7
*Aerobic fitness*	*n* = 110	*n* = 128	*n* = 238
VO_2_max (ml/kg/min)	40.3±5.5	51.5±7.4^†^	46.3±8.6
VO_2_max_FFM_ (ml/kg_FFM_/min)	58.9±5.6^a^	64.5±6.2^†^	61.9±6.5^b^
*Blood values*	*n* = 116	*n* = 129	*n* = 245
CRP (mg/L)	1.4±1.7	1.0±1.2	1.2±1.5
HOMA (mU/L)	1.6±0.8	1.9±1.4	1.8±1.2

Values are mean±SD. BMI = body mass index, VO_2_max = maximal oxygen uptake, VO_2_max_FFM_ = maximal oxygen uptake relative to the fat-free mass, CRP = C-reactive protein. Significant sex differences

*p<0.01

^†^p<0.001

^a^n = 109

^b^n = 237.

When classified based on BMI, 20.7% and 25.6% (p = 0.407) of the girls and boys, respectively, were categorized as overweight/obese, however when classified based on %fat, the proportions of over-fat girls and boys were 42.5% and 58.1% (p = 0.015), respectively. A higher percentage of boys (81.3%) were classified with high fitness than girls (54.5%, p < 0.001), whereas a greater proportion of girls (22.7%) were classified into the low fitness category compared to the boys (10.2%, p < 0.001). Most of the students had low CRP levels (<1 mg/l) or 58.6% of the girls and 75.2% of the boys, however, 12.1% of the girls and 6.2% of the boys (p = 0.028) had high levels (>3 mg/l) [40].

All adiposity measurements (WC, BMI, fat mass, aFM, and %fat) were highly correlated when adjusted for sex (r = 0.69–0.96, p < 0.001). Furthermore, all adiposity measurements, adjusted for sex, were positively related to CRP where %fat, fat mass, and aFM had the highest correlations (Table 2). The strength of these relationships held despite controlling for VO_2_max, or VO_2_max_FFM_. VO_2_max, controlled for sex, showed negative associations with the adiposity measurements (r = -0.45 to -0.71, p < 0.001), as well as with CRP (Table 2). The latter relation was independent of BMI or WC but became non-significant when controlled for DXA measures of adiposity (Table 2). In contrast, VO_2_max_FFM_ was unrelated to the adiposity measures (r = -0.10 to 0.03, p = 0.139–0.735) and CRP after adjusting for sex (Table 2). Both measures of fitness were highly related (r = 0.74, p < 0.001) after controlling for sex, however. Additional adjustment for HOMA did not change any of the above mentioned relationships, although the partial correlation coefficients became somewhat attenuated (Table 2).

**Table 2:** Relationship between measures of adiposity, fitness and CRP.

		CRP			CRP	
Variable	Controlling for*	*r*	*P*	Controlling for^†^	*r*	*P*
WC	-	**0.26**	**<0.001**	-	**0.19**	**0.004**
	VO_2_max	**0.13**	**0.041**	VO_2_max	0.13	0.054
	VO_2_max_FFM_	**0.24**	**<0.001**	VO_2_max_FFM_	**0.20**	**0.002**
BMI	-	**0.25**	**<0.001**	-	**0.19**	**0.003**
	VO_2_max	**0.14**	**0.032**	VO_2_max	**0.13**	**0.050**
	VO_2_max_FFM_	**0.24**	**<0.001**	VO_2_max_FFM_	**0.18**	**0.005**
Fat mass	-	**0.31**	**<0.001**	-	**0.24**	**<0.001**
	VO_2_max	**0.16**	**0.014**	VO_2_max	**0.14**	**0.027**
	VO_2_max_FFM_	**0.30**	**<0.001**	VO_2_max_FFM_	**0.23**	**<0.001**
aFM	-	**0.31**	**<0.001**	-	**0.24**	**<0.001**
	VO_2_max	**0.17**	**0.010**	VO_2_max	**0.15**	**0.020**
	VO_2_max_FFM_	**0.30**	**<0.001**	VO_2_max_FFM_	**0.24**	**<0.001**
%fat	-	**0.33**	**<0.001**	-	**0.26**	**<0.001**
	VO_2_max	**0.18**	**0.005**	VO_2_max	**0.17**	**0.010**
	VO_2_max_FFM_	**0.32**	**<0.001**	VO_2_max_FFM_	**0.26**	**<0.001**
VO_2_max	-	**-0.30**	**<0.001**	-	**-0.21**	**0.001**
	WC	**-0.21**	**0.001**	WC	**-0.15**	**0.022**
	BMI	**-0.21**	**0.001**	BMI	**-0.16**	**0.016**
	Fat mass	-0.12	0.075	Fat mass	-0.09	0.160
	aFM	-0.12	0.062	aFM	-0.10	0.143
	%fat	-0.08	0.221	%fat	-0.06	0.341
VO_2_max_FFM_	-	-0.10	0.142	-	-0.04	0.363
	WC	-0.10	0.112	WC	-0.06	0.344
	BMI	-0.11	0.103	BMI	-0.07	0.317
	Fat mass	-0.08	0.211	Fat mass	-0.06	0.363
	aFM	-0.08	0.204	aFM	-0.06	0.359
	%fat	-0.07	0.298	%fat	-0.05	0.428

*Sex controlled for in all correlations.

^†^ = Sex and homeostatic model assessment controlled for in all correlations. WC = waist circumference, BMI = body mass index, aFM = android fat mass, %fat = body fat percentage, VO_2_max = maximal oxygen uptake, VO_2_max_FFM_ = maximal oxygen uptake relative to the fat-free mass. See Table 1 for units.

After adjustment for sex, overweight/obese participants had higher levels of CRP across both fitness classifications (VO_2_max p = 0.021; VO_2_max_FFM_ p = 0.001) than their normal weight peers. Similarly, over-fat participants had higher values of CRP across VO_2_max_FFM_ categories (p = 0.004), but interestingly, there was no such effect across VO_2_max categories (p = 0.117). Participants classified with high VO_2_max had lower CRP levels across BMI categories (p = 0.009) but not across %fat categories (p = 0.109), and no differences were found in CRP values between VO_2_max_FFM_ categories across both fatness classifications (BMI p = 0.058, %fat p = 0.196). No interaction effects were observed between the fitness and fatness classifications (p = 0.266–0.849).

**Fig 1:**
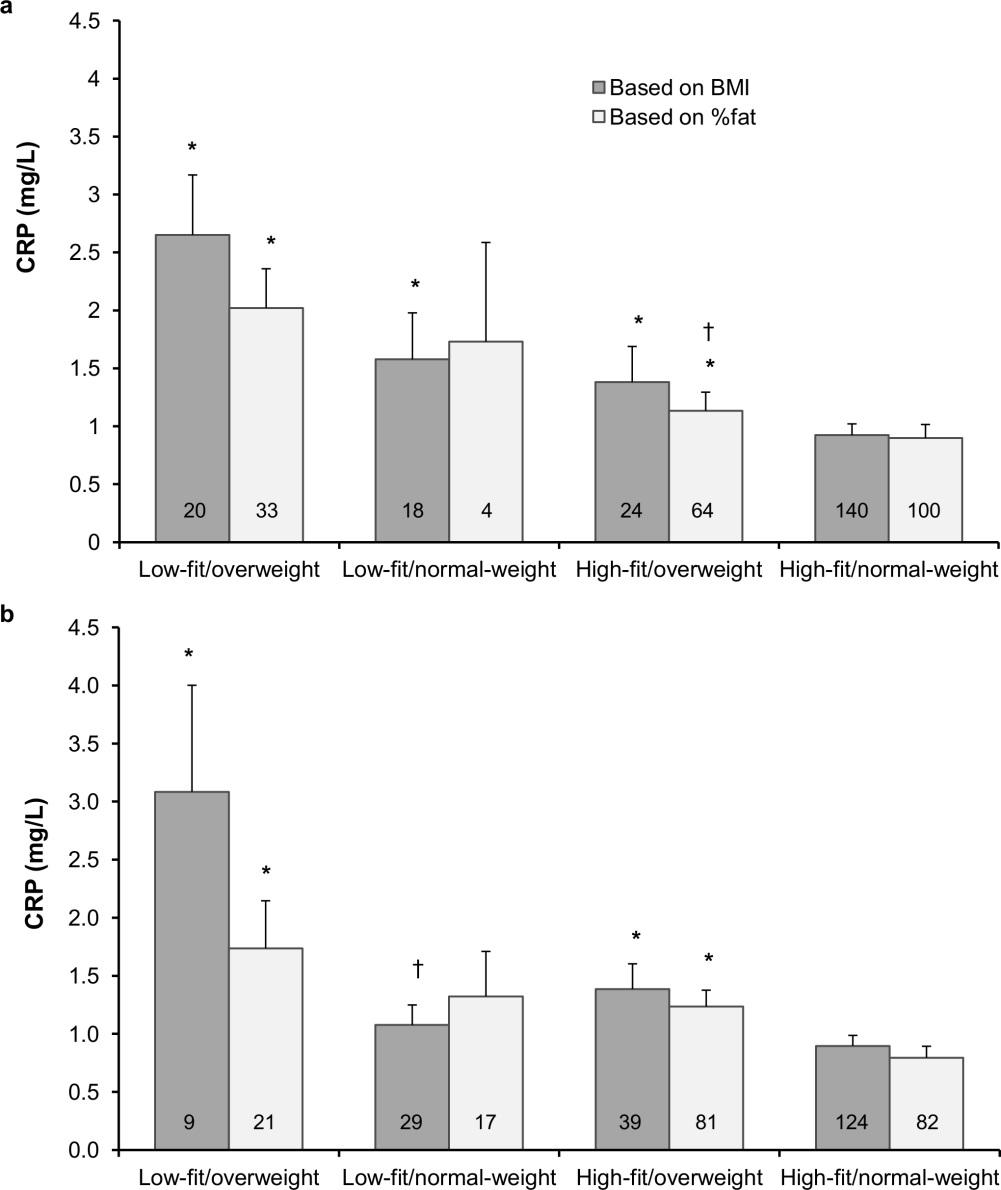
Comparison of CRP values across the fit-fat groups based on maximal oxygen uptake (a) and maximal oxygen uptake relative to the fat-free mass (b). Values are means and SE. Numbers in bars represent the number of participants in each group. CRP = C-reactive protein, BMI = body mass index, %fat = body fat percentage, *Significantly different from the high-fit/normal-weight group, *p* < 0.05. ^†^Significantly different from the low-fit/overweight group, *p* < 0.05.

CRP values across fit-fat groups are compared in Fig 1. Adolescents classified as high-fit/normal-weight based on VO_2_max and BMI had significantly lower CRP values compared to the other fit-fat groups (Fig 1A). Likewise, those classified as high-fit/normal-weight based on VO_2_max and %fat had significantly lower CRP compared to those high-fit/overweight and low-fit/overweight. Similarly, using VO_2_max_FFM_ and either BMI or %fat, the high-fit/normal-weight had significantly lower CRP both from those classified as high-fit/overweight and low-fit/overweight (Fig 1B). Using %fat and VO_2_max, the low-fit/overweight adolescents had significantly higher CRP values than their high-fit/overweight counterparts (Fig 1A) but using VO_2_max_FFM_ and BMI the low-fit/overweight adolescents had significantly higher CRP than those low fit/normal weight (Fig 1B).

## Discussion

The main objective of the study was to assess the relationship between adiposity measurements, fitness and CRP in late adolescence using direct measures of fitness and fatness. Although the results indicate that both fatness and fitness may contribute to the level of CRP in late adolescence, the major finding, contrary to our hypothesis, was that fitness adjusted for body composition (VO_2_max_FFM_) was unrelated to CRP. Our results suggest that previous findings of independent effects of fitness on CRP may have been blurred with multicolinearity due to the strong inverse relation between fitness and fatness. Furthermore, since fatness factors both in the value of fitness relative to body mass and in the value of the adiposity measure controlled for, the variables violate the statistical assumption of independence. To our knowledge, we present novel data obtained using objective methodology to study the relationships between fitness, fatness and CRP and add to the literature by analyzing the data in a manner not done previously.

Our results also suggest that the use of precise methods of body fat measurements such as DXA rather than relying on anthropometric adiposity indicators such as BMI or WC may be superior for studying the relation to CRP. Others have emphasized the importance of precise methods for estimating of both fitness and fatness [13,14].

Studies have previously examined the association of chronic low-level systematic inflammation, indicated by measures of CRP, with measures of fatness and fitness in various age-groups [12–16,18,21–23,29] although none of them have focused specifically on late adolescence as in our study. Some of the previous studies in children/adolescents have also been conducted across wide age range [13,14,22], undoubtedly affected by pubertal status, which was not always controlled for. Regardless, our results support the general agreement of these studies that increased fatness, whether indicated with BMI, WC, fat mass, %fat, or aFM, is related to higher levels of CRP.

Similarly, most of these studies [12–15,18,22,23,29] found increased unadjusted fitness, whether estimated from a submaximal test, predicted from a maximal test, or directly measured, linked to lower CRP as we found in this study. However, in many of the aforementioned studies [12–15,23] fitness and adiposity were strongly related leading to a potential problem of multicolinearity, which can both exaggerate and attenuate the independent effects of fitness. Among children and adolescents, the relations between fitness and cardiovascular risk factors were greatly influenced by the choice of expression (absolute, relative to body mass, relative to fat-free mass) of fitness [30]. Fitness_FFM_ may be the best indirect estimate of the metabolic capacity of the muscle [30] and is a way to estimate the effects of fitness adjusted for body composition. Fitness_FFM_ is also not subject to multicolinearity since it is unrelated to adiposity. In the present study, VO_2_max_FFM_ was not associated with CRP. To our knowledge, relating fitness expressed relative to the FFM to CRP has not been done previously, although such expression has lowered the relation between fitness and cardiovascular risk factors compared to expressing fitness relative to body mass [41]. The difference in the relations between VO_2_max vs. VO_2_max_FFM_ and CRP in the present study stems from the significant associations between adiposity and CRP. The use of a fitness index relative to body mass and adjusting it for adiposity is, therefore, highly questionable and may, along with indirect measures or estimates of fitness and fatness, factor in the reason for lack of agreement between studies about whether the impact of fitness on CRP is independent of fatness [12–14,16,17,23,29].

All adiposity measures retained a significant relationship with CRP when adjusted for VO_2_max although the relationship was reduced, which is similar to what others have found in adolescents [14] and adults [27,29]. However, such an adjustment is subject to the same concerns of multicolinearity and lack of independence as discussed above. Indeed, correcting the adiposity measures for VO_2_max_FFM_ had no effects on their relations to CRP. These findings confirm our hypothesis that fatness is more influential on CRP level than fitness and are supported by reports from children [12,19,23], adolescents [14], and adults [15,16,27,29]. Evidence from exercise intervention studies also suggest that fatness has a greater impact than fitness on CRP as only those exercisers who were in the highest tertile or quartile of body mass/fat loss had a significant reduction in CRP [21,24]. Similarly, exercise-induced reduction in CRP was not independent of changes in body composition [28]. This effect may depend on age, however, since among young women such an exercise-initiated decrease in CRP was independent of changes in body composition [25], and among children, the changes in CRP were not related to changes in body composition [26]. The interactive effects of fitness and fatness on CRP may also be more complicated as the effect appears to be larger among obese participants [25] and among more fit participants [28].

Fitness may indeed have important protective effects as those who were classified as high-fit/normal-weight based on empirical classifications [31,38,39] had significantly lower CRP values than the other fit-fat groups (Fig 1A). Moreover, high-fit/overweight individuals, when classified based on %fat, had significantly lower CRP values than their low-fit counterparts. This is similar to findings in adults [16,29] and children [23], although in two of these studies no difference between high-fit and low-fit overweight individuals was observed, possibly due to poor measures of fatness and fitness [16] or low number (n = 4) of high-fit/overweight participants [23]. Similarly, girls with the same baseline adiposity level had lower longitudinal increase in CRP if baseline fitness was higher [42]. Unfortunately, no empirical classifications for fitness_FFM_ exist. Using the same proportions, within sex, resulting from the empirical VO_2_max classification to classify participants into low-, average-, and high fitness_FFM_ categories along with empirical cut-offs for adiposity, really only distinguished between fatness classifications within fitness categories (Fig 1B).

Fitness and fatness can be linked to inflammation via several mechanisms. Adipocytes release proinflammatory cytokines [43], which stimulate the synthesis of CRP in the liver. High levels of free-fatty acids in the blood can also activate proinflammatory genes [44], a process modulated by exercise in animals [45]. The anti-inflammatory effects of exercise could also be mediated by increased insulin sensitivity and higher levels of high-density lipoproteins and adiponectin [43], and improvements in endothelial function may further play a role [17]. Inflammation and insulin resistance have been linked [14] and the impact of fitness and fatness on CRP might be indirect via insulin resistance. However, the present findings suggest that insulin resistance does not exert much influence on the relationship between adiposity, fitness and CRP as has been previously reported [14].

Our study was not without limitations. First and foremost is the cross-sectional design, which does not allow for the determination of a cause and effect relationships. Secondly, using young adult empirical cut-offs to classify 18 year-olds may be debatable. In addition, albeit significant, the variance independently explained in the correlational analysis was rather low (2–11%). Finally, dietary factors were not taken into account but dietary choices may contribute to inflammation. The Icelandic diet is generally high in protein (18%) and relatively low in carbohydrates (44%), whereas total fat consumption is moderate (36%), and 2% of the energy comes from alcohol [46]. Unpublished data from our research center on dietary intake of 18 year-olds reveals similar division of macronutrient but with slightly lower proportions of fat and alcohol in favour of carbohydrates (18% protein, 50% carbohydrates, 32% fat, 1% alcohol). We believe the strengths of the study overcome its limitations in that the cohort was relatively large, focused on a relatively healthy understudied population (late adolescence), and that empirical cut-offs were used to classify participants on BMI, %fat and VO_2_max. Additionally, we used precise laboratory measurements to assess fatness and fitness, which enabled us to demonstrate the association of fitness adjusted for body composition to CRP as well as the importance of considering multicolinearity when examining the interactive association of fitness and fatness to CRP levels. Neither has, to our knowledge, been done previously. Finally, the findings should have high generalizability as the cohort reasonably reflects 18 year-old adolescents in Iceland because it was recruited from metropolitan Reykjavik where 67% of all Icelandic high-school students are enrolled and all academic structures were represented.

In conclusion, our results support findings from other studies that fatness has a greater association with CRP than fitness. However, fitness_FFM_, which is truly independent of adiposity, is not related to CRP, indicating that the previously reported independent effects of fitness might be mediated via the fatness component inherently embedded in fitness when it is expressed relative to body mass. Further studies, especially in different age groups, are needed to confirm or refute our findings. Finally, our study demonstrates the importance of using precise measurements of fitness and fatness when researching the interactive effects of the variables on CRP.

## Supporting Information

S1 Dataset Raw data. (XLSX)
